# Common DNA Damage Response Factors Required for Cellular Resistance to Inhibitors for the Ataxia Telangiectasia and Rad3-Related Checkpoint Kinase in Hematopoietic Cells

**DOI:** 10.3390/biom16060851

**Published:** 2026-06-10

**Authors:** Muhammad Tufail, Ryotaro Kawasumi, Sangita Dattatray Shinde, Sivapriya Kirubakaran, Kouji Hirota

**Affiliations:** 1 Department of Chemistry, Graduate School of Science, Tokyo Metropolitan University, Minamiosawa 1-1, Hachioji-shi 192-0397, Tokyo, Japan; m.tufail@bs.qau.edu.pk (M.T.); 19_rkawa@tmu.ac.jp (R.K.); 2 Department of Chemistry, Indian Institute of Technology Gandhinagar, Palaj, Gandhinagar 382055, Gujarat, India; sangita.shinde@iitgn.ac.in

**Keywords:** DNA replication, checkpoint, DNA damage response (DDR), cancer chemotherapy, DT40, TK6

## Abstract

Targeting checkpoints is one of the most promising strategies in cancer chemotherapy. Leukemia, in particular, is expected to yield high therapeutic efficacy due to its high replication stress. However, the DNA damage response factors involved in the vulnerability to checkpoint inhibitors of these hematopoietic cancers remain elusive. In this study, we reveal common factors required for cellular resistance to ATR inhibition in hematopoietic cancer cells. We explored the DNA damage response pathways contributing to cellular tolerance to three types of ATR inhibitors using an isogenic DNA repair factor mutant collection derived from the chicken lymphoma cell line, DT40. We first demonstrated significant ATR inhibition activity of the recently developed Torin2 analogous compounds, SPK67 and SPK98, under stressed replication conditions. We then compared cellular sensitivity patterns of the known ATR inhibitor, VE-821, and the potential ATR inhibitors, SPK67 and SPK98, in 24 types of mutants deficient in genome maintenance systems and found that *RAD17*^/−^, *FEN1*^−/−^, and *POLB*^−/−^ cells exhibited hypersensitivity to all these drugs. Consistently, these mutant cells exhibited increased chromosome instability upon treatment with VE-821, SPK67, and SPK98, resulting in apoptosis. These results suggest that Rad17, Fen1, and Polymerase β play roles in responding to DNA damage caused by these drugs. However, ATR inhibition did not result in cell-cycle arrest, Chk1 phosphorylation, or increased γH2AX levels. These results suggest that, although ATR inhibition causes DNA damage, impaired checkpoint function suppresses the appropriate activation of DNA damage signaling pathways, thereby leading to cell death. This study is the first to demonstrate the importance of Rad17, Fen1, and Polymerase β in cellular tolerance to ATR inhibition in hematopoietic cells.

## 1. Introduction

Ataxia-telangiectasia and Rad3-related (ATR) checkpoint pathway plays a crucial role in maintaining the robustness of DNA replication [1]. Aberrant replication fork structure containing single-stranded (ss) DNA activates the replication stress response checkpoint, primarily mediated by the ATR kinase [2]. This checkpoint kinase mediates the replication stress signal to downstream mediators, including Chk1, via phosphorylation [3], and ultimately maintains the robustness of DNA replication through suppressing new origin firing [4,5,6] and preventing exhaustion of dNTP, and replication protein A (RPA) [7]. This checkpoint axis is also crucial for the safe replication fork arrest on a damaged DNA template [8]. Thus, the ATR axis is a major DNA damage checkpoint mediator guarding replication stability and genome integrity.

Targeting the ATR–Chk1 checkpoint axis is one of the most promising strategies in cancer chemotherapy [9]. Since ATR plays a role in preventing DNA degradation from gapped DNA and suppressing uncoupling of the forks, inhibition of ATR results in increased DNA damage in the genome [10]. Based on such DNA damage suppressive roles of ATR, several ATR inhibitors are progressing into clinical trials as promising cancer drugs [11,12]. Since, among cancers, leukemia generally possesses massive replication stress, reduced DNA damage response (DDR) ability, and abnormalities in nucleoside metabolism, use of the ATR inhibition strategy for this type of cancer is expected to yield high therapeutic efficacy [13,14]. Indeed, we found stronger cellular vulnerability to ATR inhibitors in lymphoid and myeloid leukemia compared to all other cancers in the GDSC2 dataset (DepMap Public 25Q3 release; DepMap, Broad Institute, 2025) [15,16] (Figure S1A). However, the DDR factors required for cellular resistance to ATR inhibitors in hematopoietic cell lines remain to be elucidated.

DDR pathways involve DNA damage checkpoints, DNA damage repair systems, and DNA damage tolerance systems. ATM and ATR axes are two major DNA damage checkpoints sensing DNA double-strand breaks (DSBs) and ssDNAs, respectively [17]. DNA damage sensor proteins Rad9, Rad1, and Hus1 form the 9-1-1 checkpoint clamp, which is loaded onto the 5′ junction of ssDNA by the Rad17 clamp loader, thereby initiating ATR signaling [18]. DNA damage repair systems include base excision repair (BER), nucleotide excision repair (NER), DSB repair, and crosslink repair [19,20,21,22]. BER is involved in the repair of damaged bases, such as deaminated bases and single-strand breaks (SSBs) [19,23]. NER is required for the repair of nucleotide damage that causes distortion in the DNA backbone, such as ultraviolet (UV)-induced thymidine dimers [22]. DSBs are among the most formidable DNA lesions that cause cell death if they are not repaired in a timely manner; also, homologous recombination (HR) and non-homologous end joining (NHEJ) are involved in this repair system [21]. Crosslink repair systems include systems for repairing interstrand crosslinks in DNA and DNA-protein crosslinks [20,24]. DNA damage tolerance systems rescue stalled replication by translesion DNA synthesis (TLS) and template switching mediated by HR [25].

One of the representative ATR inhibitors is VE-821, with high selectivity and efficacy in IC_50_ 26 nM [26]. The derivative of this drug with higher metabolic stability, such as VE-822 (a.k.a. VX970 or berzosertib), has demonstrated promising antitumor activity in patients in a phase II clinical trial [27,28,29] (chemical structures for VE-821 and VE-822 are shown in Figure S1B). In addition to these agents, Ceralasertib [30], Elimusertib [31], Camonsertib [32], Gartisertib [33], and Torin2 [34] are used as ATR inhibitors (Figure S1C).

These compounds inhibit ATR with a similar mechanism. ATR catalyzes the transfer of a phosphate group from its substrate, adenosine triphosphate (ATP). However, these inhibitory compounds compete with ATP for the catalytic site of ATR, and as a result, inhibit ATR activity by occupying its ATP-binding site [26]. Torin2 is an inhibitor of the phosphoinositide 3-kinase-related kinase family, including mTOR kinase, and thus this compound has a wider range of targets to inhibit than ATR inhibitors.

**Figure 1:**
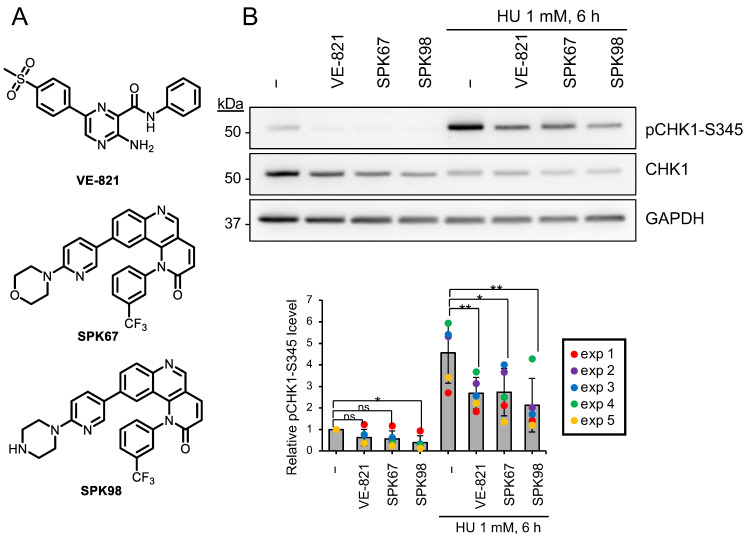
ATR inhibition by SPK67 and SPK98. (**A**) Structure of VE-821, SPK67, and SPK98. (**B**) Representative images of Western blot. Wild-type TK6 cells were incubated with 0 or 1 mM HU for 6 h in the presence of VE-821 (2.5 μM), SPK67 (1 μM), or SPK98 (500 nM). Whole cell extracts were blotted for Chk1-pS345 (Chk1-p), Chk1, and GAPDH (loading control). Data are presented as the mean ± SD from five independent experiments. Statistical significance was determined using Student’s *t*-test. ns, *p* > 0.05; * *p* < 0.05; ** *p* < 0.01.

Recently, novel ATR inhibitors were identified from analogs of Torin2 based on the structural similarity between mTOR and ATR kinases (Figure 1A) [35]. The initial characterization of such compounds revealed that SPK67 has inhibitory activity only against mTOR kinase, while SPK98 efficiently inhibits both ATR and mTOR [35]. However, ATR inhibition was assessed by monitoring phosphorylation of ATR substrates following UV irradiation at doses exceeding lethal levels, which raises concerns about the validity of this conclusion. Moreover, no direct comparison of these drugs to VE-821 regarding their cellular effects has been conducted, and thus, the efficacy of these drugs in cancer treatment has not been clarified.

To understand the role of numerous DDR factors, the chicken lymphoma DT40 cell line has been used extensively [25]. This is because this cell line is capable of gene disruption even without CRISPR, being the sole exception among animal cells [36]. As a result of extensive research over the past three decades, a vast DDR mutant collection from the DT40 cell line has been created [25]. Following the advent of the CRISPR technique, a similarly large number of mutant cells has been generated from the human lymphoblastoma TK6 cell line, leading to the creation of the collection of mutated human cells from TK6 (COMHUT) [37].

In this study, we re-evaluated SPK67 and SPK98, which were previously characterized as an mTOR inhibitor and an ATR/mTOR dual inhibitor, respectively, and found that both compounds are potent ATR inhibitors. Furthermore, we explored the DDR pathways responsible for cellular resistance to the ATR inhibitors, VE-821, SPK67, and SPK98 in the chicken DT40 cells. We identified *RAD17*^/−^, *FEN1*^−/−^, and *POLB*^−/−^ cells as the mutants that commonly show hypersensitivity to these three drugs. Here, we reveal common factors required for cellular resistance to ATR inhibition in hematopoietic cell lines.

## 2. Materials and Methods

### 2.1. DT40 Cell Culture and Cellular Sensitivity Analysis

The DT40 and TK6 cell lines used in this study are listed in Table 1. Cell culture and cellular sensitivity analyses (ATP assay) were conducted as previously described methods [38]. DT40 cells were cultured in DMEM/F-12 medium (FUJIFILM, Tokyo, Japan) supplemented with 10% heat-inactivated fetal bovine serum (FBS) (BioWest, Nuaillé, France), 2% chicken serum (Thermo Fisher Scientific, Waltham, MA, USA), 50 μM mercaptoethanol (FUJIFILM, Tokyo, Japan), L-glutamine (Nacalai Tesque, Kyoto, Japan), 50 U/mL penicillin, and 50 μg/mL streptomycin (Nacalai Tesque, Kyoto, Japan) at 39.5 °C in a humidified atmosphere with 5% CO_2_. TK6 cells were cultured in Roswell Park Memorial Institute 1640 medium (FUJIFILM, Tokyo, Japan) supplemented with sodium pyruvate (1.8 mM), L-glutamine (2 mM), penicillin (50 U/mL), streptomycin (50 μg/mL; Nacalai Tesque, Kyoto, Japan), and 10% heat-inactivated horse serum (Thermo Fisher Scientific, Waltham, MA, USA) at 37 °C in a humidified atmosphere with 5% CO_2_. For the ATP assay, actively growing cells were treated with drugs. In this assay, 96-well plates were used to treat 800 cells with the indicated concentration of drugs in 160 μL of medium, followed by incubation at 39.5 °C for 48 h for DT40 cells or at 37 °C for 72 h for TK6 cells. According to the manufacturer’s instructions, 100 μL of the cell-containing medium was added to 96-well plates, to which CellTiter-Glo (Promega, Madison, WI, USA) was added to quantify the ATP content. Fluoroskan Ascent FL (Thermo Fisher Scientific Inc., Waltham, MA, USA) was used to measure luminescence. LD_50_ values, indicating the drug concentration that reduces cellular survival by 50%, were calculated from the survival curves using the R program (version 4.5.3), which can estimate LD_50_.

### 2.2. Inhibitors Used in This Study

The ATR inhibitor, VE-821 (Selleck Chemicals, Houston, TX, USA), SPK67, and SPK98 [35] were used.

### 2.3. Chromosome Aberration Analysis

Chromosomal aberrations (CAs) were analyzed as previously described [46]. DT40 cells were treated with drugs for 24 h and with 0.1 μg/mL colcemid (Thermo Fisher Scientific, Waltham, MA, USA) for the last 2 h to arrest the cells in the M phase. The cells were pelleted by centrifugation (1200 rpm for 5 min), resuspended in 75 mM KCl (10 mL) for 13 min at room temperature, and fixed in a freshly prepared 3:1 mixture (2 mL) of methanol and acetic acid (Carnoy’s solution). The pelleted cells were resuspended in Carnoy’s solution (7 mL), dropped onto glass slides, and air-dried. The slides were stained with 5% HARLECO Giemsa Stain solution (Nacalai Tesque, Kyoto, Japan) for 10 min, rinsed with water and acetone, and dried at room temperature. The slides were examined under an ECLIPSE-Ni microscope (NIKON, Tokyo, Japan) at 1000× magnification. The chromosomes within each mitotic cell were scored.

### 2.4. Flow Cytometric Analysis of Cell-Cycle Distribution

The cells were cultured in media with or without VE-821 (Selleck Chemicals, Houston, TX, USA) for 6 h for DT40 cells, followed by labeling with 20 μM BrdU (Tokyo Chemical Industry, Tokyo, Japan) for the last 15 min. The cells were harvested and fixed overnight in 70% ethanol at 4 °C, followed by successive incubation as follows: (i) in 2N HCl and 0.5% Triton X-100 for 30 min at room temperature; (ii) in a mouse anti-BrdU antibody (1:100; Becton, Dickinson and Company, Franklin Lakes, NJ, USA) for 60 min at room temperature; (iii) in an FITC-conjugated anti-mouse antibody (1:50; Southern Biotech, Birmingham, UK) for 30 min at room temperature; (iv) in 5 μg/mL propidium iodide (Nacalai Tesque, Kyoto, Japan) in PBS. Flow cytometric analysis was performed using a BD Accuri C6 flow cytometer (Becton, Dickinson and Company, Franklin Lakes, NJ, USA).

### 2.5. Western Blotting

Western blotting was performed to detect phosphorylated Chk1 as previously described [47]. Whole-cell extracts were prepared by lysing cells directly with 1 × Laemmli buffer (50 mM Tris-HCl, 2% SDS, 10% glycerol, 100 mM dithiothreitol, and 2.5 mg/mL bromophenol blue) and boiling for 15 min. The protein samples were separated using electrophoresis on a 10–20% gradient precast gel (FUJIFILM, Tokyo, Japan) with SDS running buffer. Proteins were then transferred onto a PVDF membrane (Merck Millipore, Burlington, MA, USA) using 1 × transfer buffer (25 mM Tris-HCl, 192 mM glycine, 20% methanol, and 0.01% SDS) at 30 V overnight in a wet transfer tank (Bio-Rad Laboratories, Hercules, CA, USA). After transfer, the membranes were saturated for 30 min using PBS-T (0.05% Tween 20) containing 5% skim milk (Morinaga Milk Industry, Tokyo, Japan). After washing with PBS-T, the membranes were incubated with primary antibodies for 1 h at room temperature. Then, the membranes were washed with PBS-T and incubated with secondary antibodies for 40 min at room temperature. After washing with PBS-T, the protein signals were detected using ImmunoStarR LD (FUJIFILM, Tokyo, Japan). Primary antibodies: anti-CHK1 (1:500; sc-8408, Santa Cruz Biotechnology, Dallas, TX, USA), anti-CHK1-pS345 (1:1000; #2341, Cell Signaling Technology, Danvers, MA, USA), anti-γH2AX (mouse) antibody (05-636; Millipore, Burlington, MA, USA), and anti-GAPDH (1:1000; sc-32233, Santa Cruz Biotechnology, Dallas, TX, USA).

### 2.6. Measurement of Apoptotic Cell Fraction Using Flowcytometry

Staining of apoptotic cells with Annexin V and measurement using flowcytometry were performed as described previously [37]. An Annexin V-FITC apoptosis detection kit (15342-54, Nacalai Tesque, Kyoto, Japan) was used.

### 2.7. Measurement of γH2AX Positive Cell Fraction Using Flowcytometry

Staining of γH2AX and measurement using flowcytometry were performed as described previously [37]. The following antibodies were used: anti-γH2AX (mouse) antibody (05-636, Merck Millipore, Burlington, MA, USA) and Alexa488-conjugated anti-mouse antibody (A11001, Invitrogen, Waltham, MA, USA).

### 2.8. Statistical Analysis

All statistical analyses were conducted by paired *t*-test using Excel software (Microsoft, Redmond, WA, USA).

### 2.9. Structural Similarity Analysis

Chemical structures of ATR inhibitors were converted into Morgan fingerprints (radius = 2, 2048 bits) using RDKit [48], based on the Morgan algorithm [49]. Pairwise structural similarity was quantified using the Tanimoto coefficient [50]. The resulting similarity matrix was hierarchically clustered and visualized as a heatmap.

## 3. Results

### 3.1. ATR Inhibition by SPK67 and SPK98

Previous studies evaluated the potency of SPK67 and SPK98 as ATR inhibitors based on reduced phosphorylation of Chk1 (Chk1-p) and histone H2AX (γH2AX) following lethal-dose UV irradiation [35]. It should be noted that ATR is not directly activated by UV-induced lesions, but rather by the replication stress that arises when unrepaired damage interferes with DNA replication [51]. In addition, γH2AX is commonly associated with DNA double-strand breaks, for which ATM is the predominant kinase [52]. Therefore, the reduction in γH2AX after UV irradiation may not specifically reflect ATR inhibition, necessitating a more careful evaluation of whether SPK67 and/or SPK98 selectively inhibit ATR, or also affect other PI3K-related kinases such as mTOR. To address this, we examined the effects of these drugs on the phosphorylation status of Chk1 (Chk1-p) under hydroxyurea (HU) treatment, which depletes the cellular dNTP pool and thereby interferes with replication [53] (Figure 1B). We found that, in addition to the well-characterized ATR inhibitor VE-821, both SPK67 and SPK98 inhibited the phosphorylation of Chk1, indicating that these compounds inhibit ATR.

### 3.2. Comparison of VE-821, SPK67, and SPK98 on the Cellular Effects

**Figure 2:**
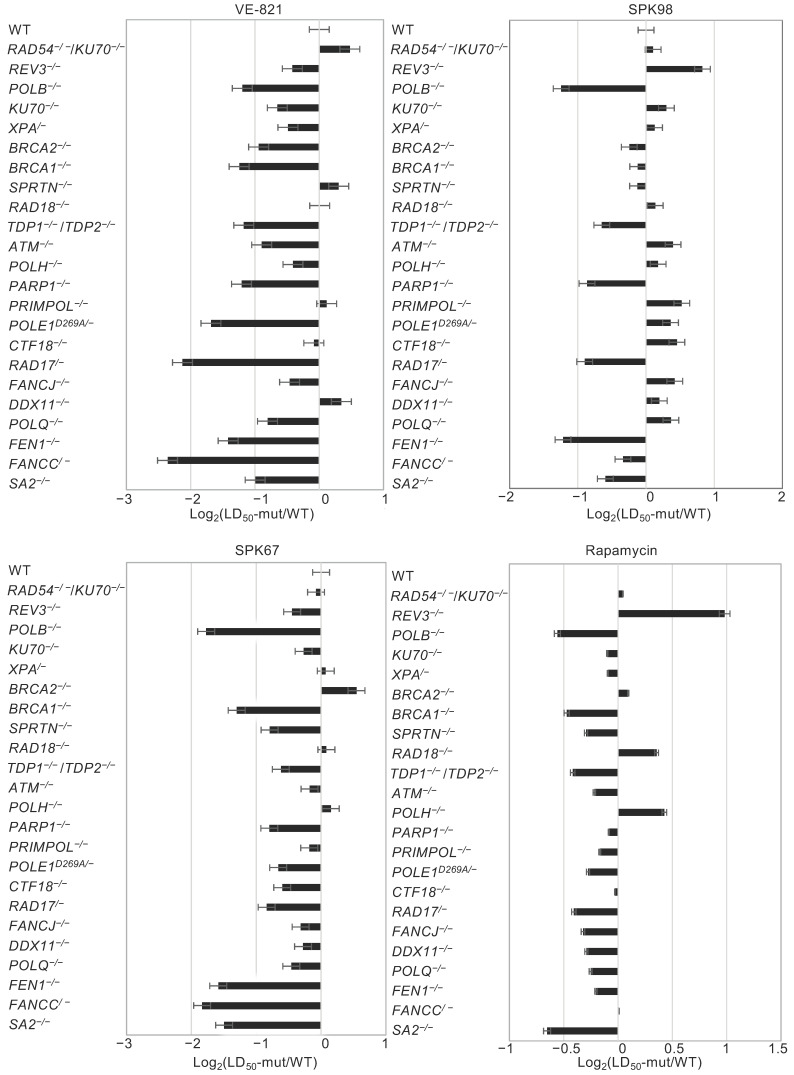
Sensitivity profiles of VE-821, SPK67, and SPK98 in the selected DT40 mutant cells. The relative sensitivity of each mutant to VE-821, SPK67, and SPK98, compared with wild-type DT40 cells, was scored as log_2_ (LD_50_ in indicated mutant cells)/(LD_50_ in wild-type cells). In this formula, LD_50_ indicates the drug concentration that reduces cell survival to 50% relative to that of untreated cells. As a result, negative (left) and positive (right) scores show that the indicated gene-disrupted cells are sensitive and resistant to the indicated drugs, respectively.

To compare the cellular effects of the potential ATR inhibitors SPK67 and SPK98 with those of the known ATR inhibitor VE-821, we attempted to identify DDR factors required for cellular tolerance to these compounds. To this end, we explored mutant cell lines showing hypersensitivity to these compounds from our mutant cell line collection generated from the chicken lymphoma DT40 cells, including mutants deficient in TLS (*REV3*^−/−^, *RAD18*^−/−^, *POLH*^−/−^), HR (*BRCA1*^−/−^, *BRCA2*^−/−^), NHEJ (*POLQ*^−/−^, *KU70*^−/−^), HR and NHEJ (*RAD54*^−/−^/*KU70*^−/−^), checkpoint signaling (*ATM*^−/−^, *RAD17*^/−^), BER (*PARP1*^−/−^, *FEN1*^−/−^, *POLB*^−/−^), NER (*XPA*^/−^), the Fanconi anemia pathway (FA; *FANCC*^/−^, *FANCJ*^−/−^), proofreading (*POLE1^D269A^*^/−^), removal of topoisomerase I-DNA cleavage complexes (Top1-cc repair; *TDP1^−/^^−^*/*TDP2*^−/−^), DNA-protein crosslink repair (*SPRTN*^−/−^), repriming of stalled replication (*PRIMPOL*^−/−^), and sister chromatid cohesion (SCC; *DDX11*^−/−^, *CTF18*^−/−^, *SA2*^−/−^) (Table 1). We assessed cellular sensitivity to VE-821, SPK67, and SPK98 using the ATP assay. We also assessed sensitivity to the well-established mTOR inhibitor, rapamycin, as a control [54]. From these sensitivity data, we calculated the relative sensitivity using the formula log_2_ (LD_50_ in the indicated mutant cells/LD_50_ in the wild-type cells) (Figure 2), where LD_50_ indicates the drug concentration that reduces cellular survival by 50%. Based on these data, we defined the mutants exhibiting a relative sensitivity value below −0.8 for all three drugs as the mutants deficient in common factors required for the cellular resistance to these agents. Using these criteria, we identified mutants deficient in checkpoint signaling (*RAD17*^/−^) and BER (*FEN1*^−/−^, *POLB*^−/−^) that commonly exhibited hypersensitivity to VE-821, SPK67, and SPK98. Among these compounds, SPK67 and SPK98 showed the most similar sensitivity pattern (Figure S2A), presumably because these compounds were derived from a common parent scaffold [35]. Indeed, SPK67 and SPK98 have the highest structural similarity, while VE-821 and VE-822 are clustered in another group (Figure S2B). Comparing SPK67 and SPK98, the sensitivity profile pattern of SPK67 was much more similar to that of VE-821, since in the case of SPK67 and VE-821, *BRCA1*^−/−^ and *FANCC*^/−^ cells were common hypersensitive mutants, but in the case of SPK98, these mutant cells did not exhibit hypersensitivity (Figure 2). Importantly, all tested mutants did not show significant augmentation in cellular sensitivity to rapamycin (relative sensitivity value < −0.8), indicating that the observed hypersensitivity is attributable to inhibition of ATR rather than mTOR kinase (Figure 2).

### 3.3. BER Pathway Is Pivotal for the Cellular Tolerance to ATR Inhibitors

**Figure 3:**
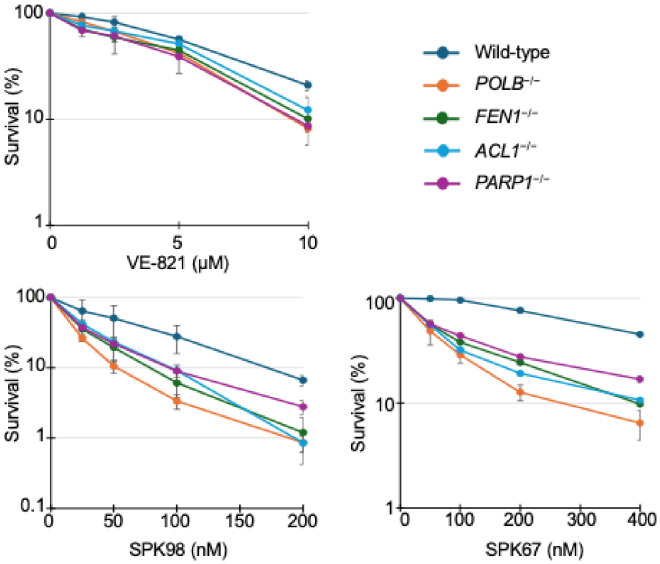
Functionality of the BER pathway is pivotal for cellular tolerance to VE-821, SPK67, and SPK98. Cellular survival (%) in the indicated DT40 cells was assessed for VE-821, SPK67, and SPK98 as described in Section 2. Cells were cultured for 48 h in the presence of the indicated concentration of drugs. Error bars represent the standard deviation (SD) from three independent experiments.

Having identified Rad17, Fen1, and Polymerase β (Polβ) as common factors required for cellular resistance to ATR inhibitors, we next inquired whether Fen1 and Polβ play roles in cellular tolerance to these drugs via BER. To this end, we compared cellular sensitivity to VE-821, SPK67, and SPK98 in BER-deficient *ALC1*^−/−^, *FEN1*^−/−^, *PARP1*^−/−^, and *POLB*^−/−^ cells with parental wild-type DT40 cells (Figure 3). We found that all tested mutants showed significantly higher sensitivity to VE-821, SPK67, and SPK98 than did wild-type cells (Figure 3). These data indicate that BER functionality is important for cellular resistance to VE-821, SPK67, and SPK98.

### 3.4. Rad17, Fen1, and Polβ Are Required for Preventing Apoptosis upon ATR Inhibitors

**Figure 4:**
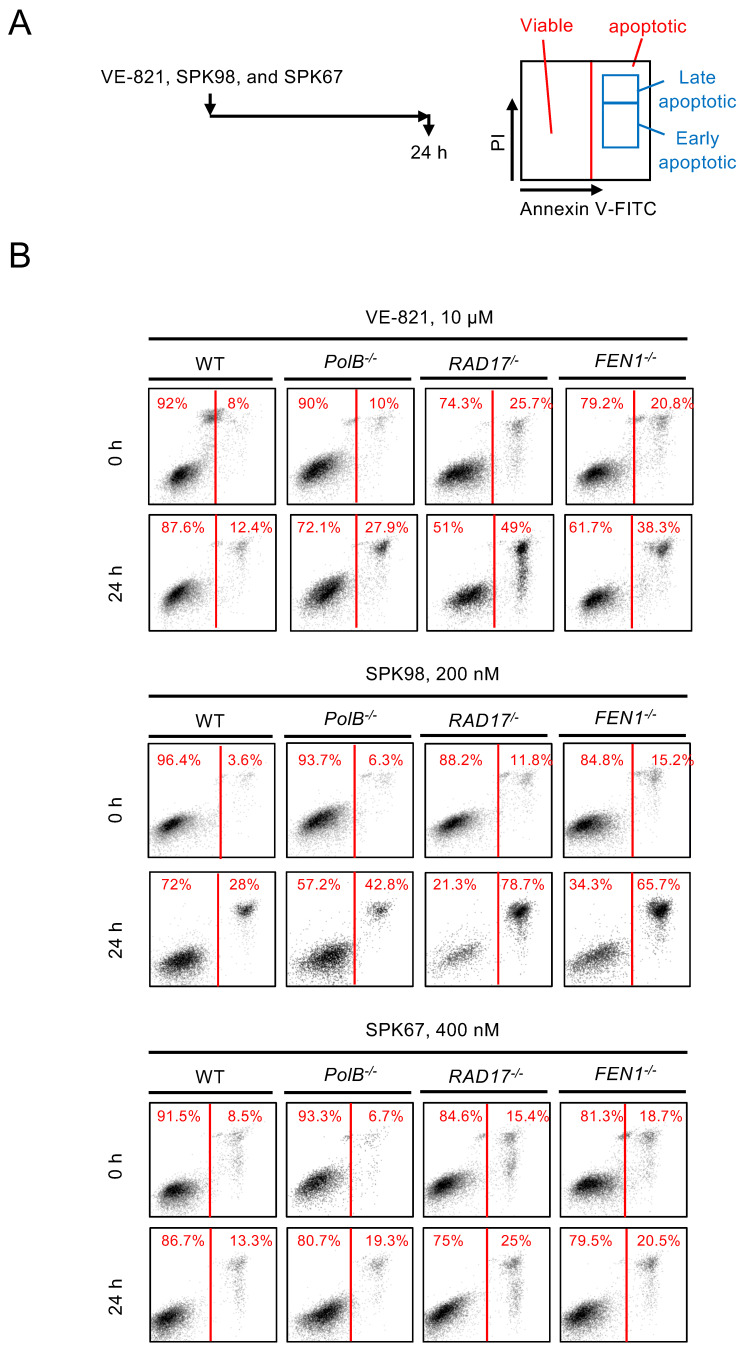
Increased apoptosis in mutant cells deficient in Rad17, Fen1, and Polβ after treatment with VE-821, SPK67, or SPK98. (**A**) The proportions of early and late apoptotic cells after treatment with 10 μM VE-821, 400 nM SPK67, or 200 nM SPK98 for 24 h were measured by Annexin V staining as described in Materials and Methods. (**B**) Annexin V staining is shown on the x-axis on a log scale, and propidium iodide (PI) staining is shown on the y-axis on a log scale. The two gates represent viable cells (lower left), early apoptotic cells (lower right), and late apoptotic cells (upper right). The numbers shown in each gate indicate the percentage of cells within that gate relative to the total analyzed cell population after excluding debris and extremely small cells. A total of 10,000 cells were analyzed per sample. The experiment was performed independently twice, and similar trends were observed in both replicates.

To investigate whether the augmented sensitivities to these compounds in *RAD17*^/−^, *FEN1*^−/−^, and *POLB*^−/−^ cells are attributable to enhanced apoptosis, we analyzed populations of the early and late apoptotic cells using Annexin V staining (Figure 4A). We found that the proportions of early and late apoptotic cells were increased in *RAD17*^/−^, *FEN1*^−/−^, and *POLB*^−/−^ cells compared to wild-type cells at 24 h after treatment with these compounds (Figure 4). Taken together, these results indicate that *RAD17*^/−^, *FEN1*^−/−^, and *POLB*^−/−^ mutations confer vulnerability to ATR inhibition.

### 3.5. Rad17, Fen1, and Polβ Are Required for Suppressing Chromosome Aberrations upon ATR Inhibitors

**Figure 5:**
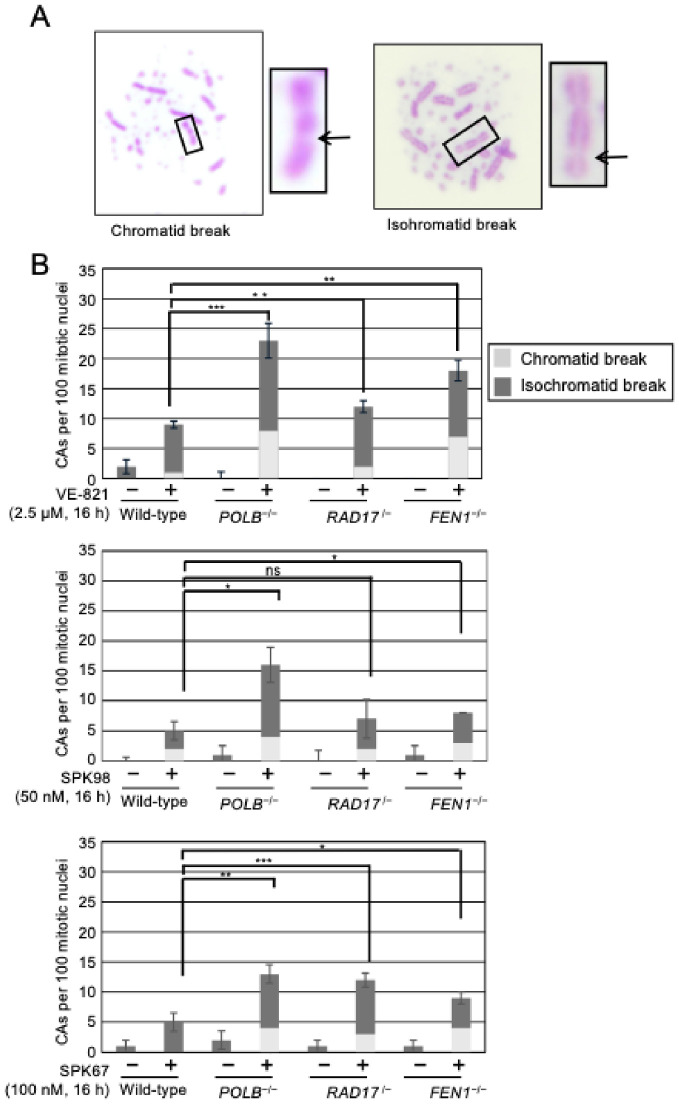
Rad17, Fen1, and Polβ contribute to maintaining the stability of the chromosome after treatment with VE-821, SPK67, or SPK98. (**A**) Representative image of chromatid break and isochromatid break in DT40 cells. Arrows indicate the break point. (**B**) Indicated cells were treated with 2.5 μM VE-821, 100 nM SPK67, or 50 nM SPK98 for 16 h. Chromosome spread and Giemsa staining were performed as described in Materials and Methods. Number of chromosomal aberrations (CAs)/100 mitotic nuclei in the indicated genotypes. Error bars represent SD from independent three tests. At least 100 mitotic cells were counted for each cell line. All statistical analyses were performed using Student’s *t*-test; * *p* < 0.05, ** *p* < 0.01, *** *p* < 0.001, ns *p* > 0.05.

Next, we investigated whether the increased cell death (apoptosis) in *RAD17*^/−^, *FEN1*^−/−^, and *POLB*^−/−^ cells during treatment with VE-821, SPK67, or SPK98 was attributable to chromosome instability. To this end, we employed a chromosome spread assay to evaluate the number of chromosome aberrations (CAs) during treatment with VE-821, SPK67, or SPK98. We measured the number of CAs and classified them into isochromatid breaks (breakage of two sister chromatids at the same location) and chromatid breaks (breakage of one of the two sister chromatids) (Figure 5A). Consistent with the sensitivity data, *RAD17*^/−^, *FEN1*^−/−^, and *POLB*^−/−^ cells exhibited an increased number of CAs after treatment with VE-821, SPK67, or SPK98 (Figure 5B). These data suggest that these potential ATR inhibitors cause DNA damage and that Rad17, Fen1, and Polβ are required for efficient repair of this damage.

### 3.6. Dysfunction of the DNA Damage Response Checkpoint Caused by ATR Inhibitors

**Figure 6:**
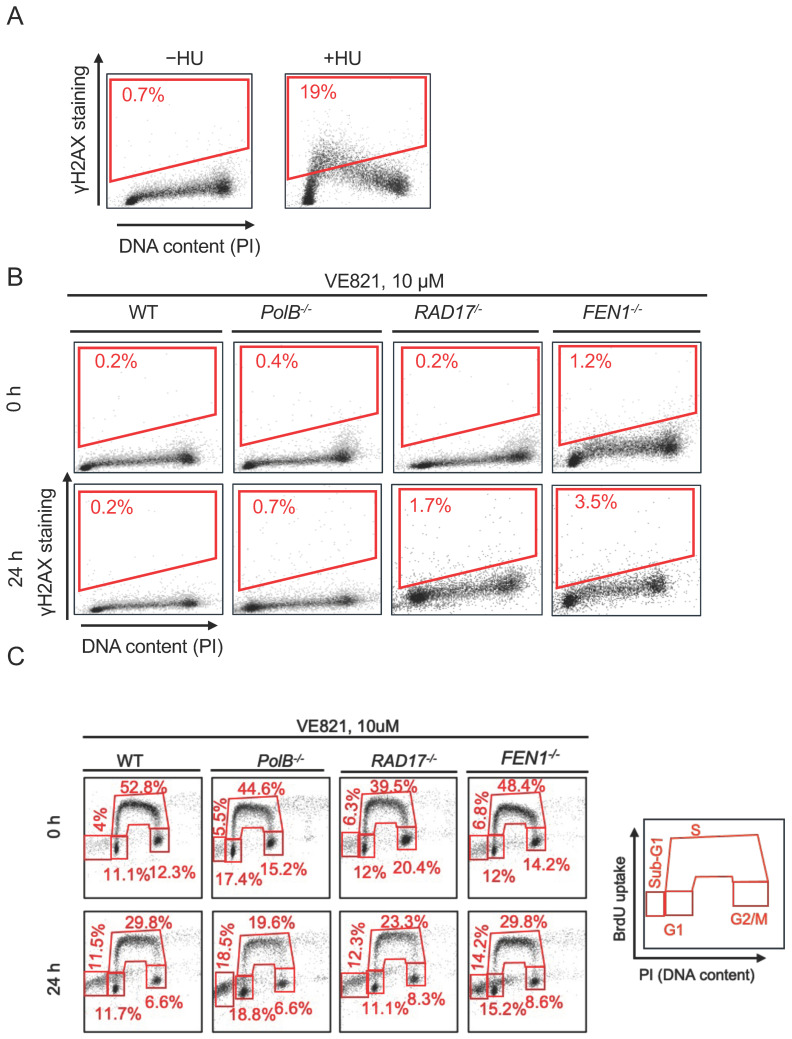
Checkpoint inactivation caused by ATR inhibition by VE-821. (**A**,**B**) Phosphorylation of Histone H2AX is not induced by VE-821 treatment. As a positive control, the proportion of phosphorylated Histone H2AX (γH2AX)-positive cells upon HU treatment (2 mM) for 1 h was measured (**A**). To assess the effect of ATR inhibition, indicated cells were treated with 10 μM VE-821 for 24 h (**B**). γH2AX staining was performed as described in Materials and Methods. After excluding cell clusters consisting of two or more attached cells, more than 7500 cells were analyzed. The percentage of γH2AX-positive cells was calculated based on the total number of analyzed cells. DNA content (stained with propidium iodide) is shown on the x-axis on a linear scale, and γH2AX levels (stained with anti-γH2AX antibody) are shown on the y-axis on a linear scale. The red gate indicates γH2AX-positive cells. The experiment was performed twice independently. (**C**) Cell-cycle distribution was analyzed as described in Materials and Methods. Indicated cells were treated with 10 μM VE-821 for 0 or 24 h. The DNA content (stained using propidium iodide) is displayed on the x-axis on a linear scale, and BrdU uptake is displayed on the y-axis on a logarithmic scale. The upper, lower most left, lower left, and lower right gates correspond to cells in the Sub-G1 (Dead cells), S, G1, and G2/M, respectively. Red numbers represent the percentage of cells falling within each gate. The proportion of cells was calculated based on the total number of cells summed across the G1, S, and G2/M gates.

To further investigate the cellular role of these DDR factors upon ATR inhibition, we analyzed the γH2AΧ signal, as γH2AΧ is induced by replication stress and DSBs. We measured the level of γH2AΧ in *RAD17*^/−^, *FEN1*^−/−^, and *POLB*^−/−^ cells after VE-821 treatment. In the positive control, we treated cells with HU. We found a strong γH2AΧ signal after HU treatment (Figure 6A), while VE-821 treatment had a limited impact on the γH2AΧ signal in all tested mutants (Figure 6B). Considering the significant increase in CAs in VE-821-treated *RAD17*^/−^, *FEN1*^−/−^, and *POLB*^−/−^ cells, we hypothesized that even after DNA damage is induced, phosphorylation of histone H2AX is not efficiently promoted due to inhibition of the ATR–Chk1 checkpoint axis by VE-821. To test this possibility, we investigated the activation status of the ATR–Chk1 checkpoint axis by monitoring the status of the phosphorylated Chk1 (Chk1-p). We found that the Chk1-p signal was indistinguishable before and after treatment with VE-821 in all tested cells (Figure S3). Taken together, these data suggest that VE-821 treatment induces more CAs in *RAD17*^/−^, *FEN1*^−/−^, and *POLB*^−/−^ cells than in wild-type cells without activating the ATR–Chk1 checkpoint axis. Consistently, VE-821 treatment did not cause accumulation at specific cell-cycle stages due to the checkpoint-mediated cell-cycle arrest at G1 or G2 (Figure 6C). Instead, cells in the S phase were reduced, and cells in sub-G1, reflecting the dead cell fraction, were increased (Figure 6C). The degree of sub-G1 augmentation was more pronounced in *RAD17*^/−^, *FEN1*^−/−^, and *POLB*^−/−^ cells, which supports increased apoptosis in these cells (Figure 4B). Thus, ATR inhibition might induce both DNA damage and checkpoint deficiency, resulting in cell death.

### 3.7. Important Role of Rad17, Fen1, and Polβ in the Cellular Tolerance to ATR Inhibitors in Human Cells

**Figure 7:**
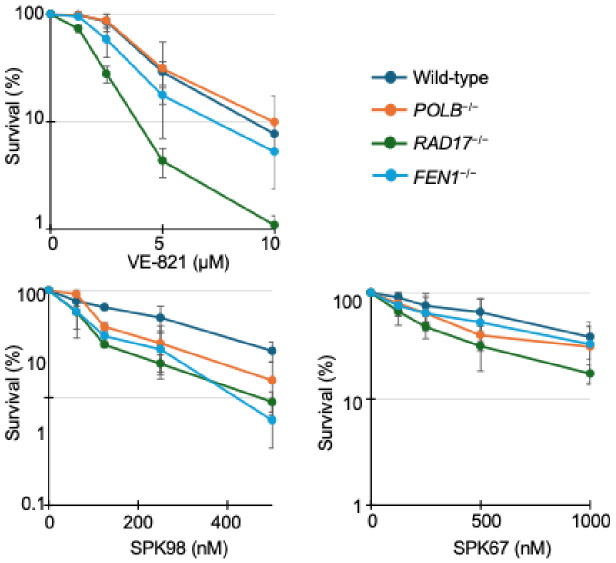
Conserved DDR factors commonly required for the cellular tolerance to ATR inhibitors in human lymphoblastoma TK6 cells. Indicated TK6 cells were treated with VE-821, SPK67, or SPK98 for 72 h. Cellular survival (%) was measured using CellTiter-Glo as described in Materials and Methods. Error bars represent the standard deviation (SD) from two independent experiments.

Finally, we investigated whether the conclusion that Rad17, Fen1, and Polβ are required for cellular tolerance to ATR inhibition in hematopoietic cells is also conserved in human systems. To this end, we employed the human lymphoblastoma TK6 cell line. We compared cellular sensitivity to VE-821, SPK67, or SPK98 between wild-type, *RAD17*^−/−^, *FEN1*^−/−^, and *POLB*^−/−^ TK6 cells (Figure 7). Similar to DT40 mutant cells deficient in Fen1 or Rad17, corresponding TK6 mutants exhibited augmented cellular sensitivity to VE-821, SPK67, or SPK98 compared to wild-type cells (Figure 7). However, the detected sensitivity was much weaker in this cell line than in DT40. Moreover, TK6 mutant cells deficient in Polymerase β showed hypersensitivity to SPK67 and SPK98 but not to VE-821. These data support the conclusion that Rad17, Fen1, and Polβ are primarily involved in maintaining cellular survival upon inhibition of ATR in human hematopoietic cells (see Section 4).

## 4. Discussion

In this study, we compared the hematopoietic cellular effects of the potential ATR inhibitors VE-821, SPK67, and SPK98 by analyzing the sensitivity profiles of a panel of DDR factor-deficient mutants generated from the chicken lymphoma DT40 cells. We found that Rad17, Fen1, and Polβ are commonly required for cellular resistance to these tested compounds (Figure 2). Since we found that all tested DT40 mutants deficient in the BER pathway also exhibited hypersensitivity to these drugs (Figure 3), the functionality of BER might be vital for the cellular tolerance to these ATR inhibitors. As these compounds induced CAs and apoptosis more pronouncedly in *RAD17*^/−^, *FEN1*^−/−^, and *POLB*^−/−^ cells (Figure 4 and Figure 5), the Rad17-mediated checkpoint axis and BER might be required for the proper processing of DNA damage caused by these compounds. However, induction of γH2AX (DNA damage marker), Chk1-p, or cell-cycle arrest was not properly induced by VE-821 treatment (Figure 6 and Figure S3), suggesting that concurrent effects of ATR inhibition, such as DNA damage induction and checkpoint failure, result in cell death. Finally, we confirmed that our current conclusion that Rad17, Fen1, and Polβ are common DDR factors involved in cellular resistance to ATR inhibitors is basically conserved in human hematopoietic cell lines (Figure 7). The data obtained here using hematopoietic cell lines might be a significant addition to our understanding of ATR inhibitors, in addition to the previously conducted comprehensive CRISPR screening using human HCT116, HeLa, and RPE1-hTERT cells [55].

We found that VE-821, SPK67, and SPK98 similarly interfere with phosphorylation of Chk1 under 1 mM HU treatment conditions (Figure 1). VE-821, SPK67, and SPK98 were tested at various concentrations, with VE-821 at the highest concentration and SPK98 at the lowest, and similar inhibitory effects were observed (Figure 1, VE-821 (2.5 μM), SPK67 (1 μM), and SPK98 (500 nM)). Consistently, obvious differences were observed in the LD_50_ values of wild-type cells for VE-821, SPK67, and SPK98, with VE-821 having the highest value and SPK98 having the lowest (Figure 3). These results suggest that SPK98 has the highest quality/intensity of inhibition activity upon ATR.

ATR inhibition induces abnormalities in DNA replication through excessive origin firing and exhaustion of the replication-related resources, such as dNTP, RPA, and PCNA, leading to the accumulation of single-strand breaks (SSBs) and gaps [8,10,56,57,58]. Moreover, such effects of the ATR inhibitors might be pronounced in hematopoietic cells due to their rapid proliferation and active DNA replication [59,60]. The BER pathway plays a critical role in SSB repair [19,23,61,62], and thus, BER factors might be pivotal for maintaining cellular tolerance to ATR inhibitors.

We also identified Rad17 as an important DDR factor for the cellular tolerance to ATR inhibitors. Previous studies have revealed the synergistic effect of ATM deficiency with ATR inhibition on cellular viability [63,64,65,66]. However, our current study did not detect a reduction in cellular survival in *ATM*^−/−^ cells upon treatment with ATR inhibitors (Figure 2). Instead, we detected a synergistic reduction in cellular viability in *RAD17*^/−^ cells. This might be attributable to the preferential augmentation of DNA replication stress rather than DSBs in hematopoietic cells under ATR-inhibited conditions. Generally, Rad17 is known not only as a sensor in the ATR checkpoint axis but also to play a key role in translating ATR activation into a checkpoint signal [67]. However, the current study suggests that the Rad17 checkpoint axis is also a critical backup for the ATR checkpoint axis, as evidenced by the observation that loss of Rad17 in ATR-inhibited conditions has a synergistic impact on cellular viability (Figure 2 and Figure 7). This view is supported by a former study showing that recruitment of Rad17 and ATR to DNA damage sites is largely independent [68].

The current study demonstrated that ATR inhibitors, VE-821, SPK67, and SPK98, cause the induction of DNA damage and inhibition of DNA damage checkpoint activation. Consistently, a former study demonstrated that the induction of extensive DNA damage (detected with alkaline comet) and significant defects in DNA damage checkpoint activation are caused by the treatment with an ATR inhibitor [69,70]. Similar to the previous study, we here observed reduced γH2AX levels while showing increased chromosome aberrations. This discrepancy might stem from a less active ATR checkpoint upon ATR inhibition, even when DNA damage is induced by ATR inhibitors. This idea is consistent with the result showing hypersensitivity to the ATR inhibitor in Rad17-deficient cells. Given the compensatory roles of Rad17 in the DNA damage checkpoint under ATR inhibition, the loss of Rad17 might have critical effects on cellular viability.

Using a DT40 mutant panel, we systematically identified Rad17, Fen1, and Polβ as factors involved in cellular tolerance to ATR inhibition. This phenotype was conserved in human TK6 cells, although the magnitude of sensitivity was less pronounced (Figure 7). This difference may reflect intrinsic properties of DT40 cells, such as elevated replication stress driven by c-Myc overexpression or the loss of the functional p53 checkpoint axis [71,72]. Moreover, TK6 cells deficient in Polβ were less sensitive to VE-821. This discrepancy might stem from differences in the usage of polymerases in chicken and human cells. The active compensation by Polymerase λ might significantly mask the requirement for Polβ in this cell line [73,74].

We identified BRCA1 and FANCC as common DDR factors required for cellular resistance to VE-821 and SPK67 but not to SPK98 (Figure 2). These factors are required for HR [75,76]. Since the involvement of HR for maintaining cellular survival upon ATR inhibition was reported [77], these factors might contribute to activating HR upon treatment with VE-821 or SPK67. However, mutants deficient in these DDR factors did not show hypersensitivity to SPK98 (Figure 1). It is possible that VE-821 or SPK67 might have a wider inhibitory effect than SPK98, and potential concurrent inhibitions of other PIKK family kinases(s) cause this difference. Considering these data, we speculate that SPK98 has the highest selectivity for ATR in comparison to VE-821 and SPK67, and potential off-target effects of VE-821 and SPK67 should be carefully considered. Although ATR has a wide range of targets, in this study, we detected only Chk1 phosphorylation to evaluate the efficiency of ATR inhibition. A wider range of targets might be needed to evaluate the specificity of ATR in our future study.

In the initial evaluation of SPK67, the inhibitory effect of SPK67 on ATR was not detected since UV-induced Chk1-p was not affected even at 1 μM of SPK67 in human HCT116 cells [35]. Meanwhile, in this study, we demonstrated that SPK67 exhibits significant inhibitory activity against the phosphorylation of ATR substrates, Chk1, and histone H2AX (Figure 1). This discrepancy might be attributed to the weaker inhibitory effect of SPK67 compared to SPK98 [35], and to the excessive UV irradiation dose (500 J/m^2^) used for the former Chk1-p assessment, which is approximately 100 times the LD_50_ for human cells [78].

## 5. Conclusions

In this study, we identified the commonly required DDR factors for cellular tolerance to ATR inhibitors in the chicken and human hematopoietic cell lines DT40 and TK6, respectively. Here, we identified Rad17, Fen1, and Polβ in our initial screening as DDR factors strongly required for maintaining cellular viability upon treatment with VE-821, SPK67, or SPK98. A limitation of this study is that it does not account for the pharmacokinetics of the inhibitory compounds. Therefore, the validity of these findings must be verified through future animal studies. The knowledge obtained in the current study will provide valuable insights for novel therapies based on the reduction of DDR activity caused by gene mutations in leukemia.

## Figures and Tables

**Table 1:** List of cell lines used in this study.

Genotype	Parental Cell Line	Function	References
Wild-type	DT40		[36]
*BRCA1* ^−/−^	Wild-type DT40 cells	HR	[39]
*BRCA2* ^−/−^	Wild-type DT40 cells	HR	[39]
*POLQ* ^−/−^	Wild-type DT40 cells	NHEJ	[39]
*KU70* ^−/−^	Wild-type DT40 cells	NHEJ	[39]
*RAD54* ^−/−^ */KU70* ^−/−^	Wild-type DT40 cells	HR/NHEJ	[39]
*POLB* ^−/−^	Wild-type DT40 cells	BER	[39]
*PARP1* ^−/−^	Wild-type DT40 cells	BER	[39]
*FEN1* ^−/−^	Wild-type DT40 cells	BER	[39]
*XPA* ^/−^	Wild-type DT40 cells	NER	[39]
*FANCC* ^/−^	Wild-type DT40 cells	Fanconi Anemia	[39]
*FANCJ* ^−/−^	Wild-type DT40 cells	Fanconi Anemia	[39]
*SPRTN* ^−/−^	Wild-type DT40 cells	TLS/Protein-DNA repair	[39]
*REV3* ^−/−^	Wild-type DT40 cells	TLS	[39]
*RAD18* ^−/−^	Wild-type DT40 cells	TLS	[39]
*POLH* ^−/−^	Wild-type DT40 cells	TLS	[39]
*PRIMPOL* ^−/−^	Wild-type DT40 cells	Repriming	[39]
*POLE1^exo^* ^−/−^	Wild-type DT40 cells	Removal of nucleoside analogs	[39]
*TDP1* ^−/−^ */TDP2* ^−/−^	Wild-type DT40 cells	Protein tyrosyl-DNA repair	[39]
*ATM* ^−/−^	Wild-type DT40 cells	Checkpoint	[39]
*RAD17* ^/−^	Wild-type DT40 cells	Checkpoint	[39]
*DDX11* ^−/−^	Wild-type DT40 cells	Cohesion	[39]
*CTF18* ^−/−^	Wild-type DT40 cells	Removal of nucleoside analogs	[39]
*SA2* ^−/−^	Wild-type DT40 cells	Cohesion	[39]
*ALC1* ^−/−^	Wild-type DT40 cells	BER	[40]
*XRCC1* ^−/−^	Wild-type DT40 cells	BER	[41,42]
Wild-type	TK6		[43]
*RAD17* ^−/−^	Wild-type TK6 cells	Checkpoint	[44]
*POLB* ^−/−^	Wild-type TK6 cells	BER	[45]
*FEN1* ^−/−^	Wild-type TK6 cells	BER	[42]

## Data Availability

All data are in the manuscript and/or Supplementary Materials. All raw data gained in this study are shown in the File S1.

## Supplementary Materials

The following supporting information can be downloaded at: https://www.mdpi.com/article/10.3390/biom16060851/s1, Figure S1: Stronger cellular vulnerability in lymphoid and myeloid than that in other cancers; Figure S2: Comparison of sensitivity profiles on 3 ATR inhibitors; Figure S3: An ATR inhibitor VE-821 does not induce phosphorylation of Chk1; Figure S4: Uncropped images used in this study; File S1: Raw data used in this study. References [79,80] are cited in the Supplementary Materials.

## Abbreviations

The following abbreviations are used in this manuscript:

ATR	Ataxia telangiectasia and Rad3-related
ATM	Ataxia telangiectasia mutated
Chk1	Checkpoint kinase 1
HU	Hydroxyurea
