# Supplementary material for: Common DNA Damage Response Factors Required for Cellular Resistance to Inhibitors for the Ataxia Telangiectasia and Rad3-Related Checkpoint Kinase in Hematopoietic Cells
Source: Biomolecules. 2026 Jun 10;16(6):851. doi: 10.3390/biom16060851 (PMC13296418; doi:10.3390/biom16060851)
Supplement: Supplementary file 1 [file biomolecules-16-00851-s001.zip › biomolecules-4342905 proof re Supplementary Fugures.pdf]

A

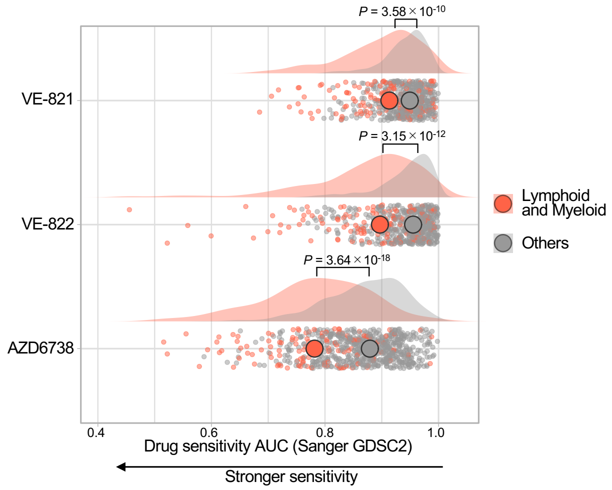

B

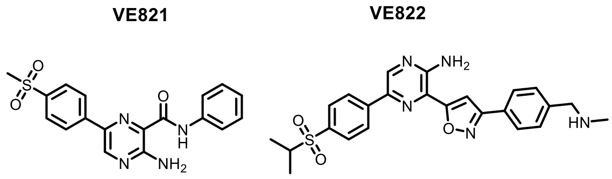

C

Compound	Other names	Inhibition mechanism
VE821	—	Competition with ATP
VE822	VX-970, M6620, Berzosertib	Competition with ATP
Ceralasertib	AZD6738	Competition with ATP
Elimusertib	BAY1895344	Competition with ATP
Camonsertib	RP-3500	Competition with ATP
Gartisertib	VX-803, M4344	Competition with ATP
Torin2	—	Competition with ATP

Figure S1 Stronger cellular vulnerability in lymphoid and myeloid than that in other cancers (A) Cancer cell lines of lymphoid and myeloid origin were separated from other cell types and plotted using SuperPlotsOfData [79]. The x-axis represents the area under the dose–response curve (AUC), which indicates sensitivity to the indicated ATR inhibitors. Filled circles represent individual cell lines, whereas black-outlined circles denote the median. Drug sensitivity data were obtained from the GDSC2 dataset (DepMap Public 25Q3 release; DepMap, Broad Institute, 2025) [15,16]. Statistical significance was assessed using Welch's *t*-test. (B) Structures of VE-821 and VE-822. (C) List of ATR inhibitors.

A

	VE821	SPK98	SPK67
VE821	1	0.53607818	0.5937109
SPK98	0.53607818	1	0.6108814
SPK67	0.5937109	0.6108814	1

B

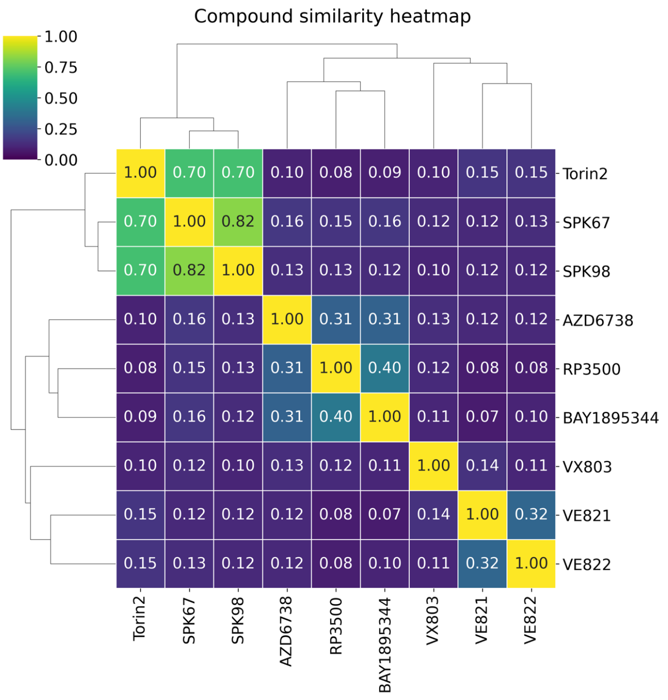

Figure S2 Comparison of sensitivity profiles on 3 ATR inhibitors

(A) From the pattern of sensitivity (which mutant shows hypersensitivity) of nucleoside analogs, pairwise Pearson correlation coefficients were calculated using the “Correlation” tool in the Data Analysis ToolPak of Microsoft Excel. The score 1.0 represents the same sensitivity profile. (B) The heatmap displays pairwise Tanimoto similarity scores between ATR inhibitors. Color intensity represents the degree of structural similarity, with values ranging from 0 to 1; values closer to 1 indicate greater structural similarity. Rows and columns are hierarchically clustered based on pairwise similarity relationships among compounds.

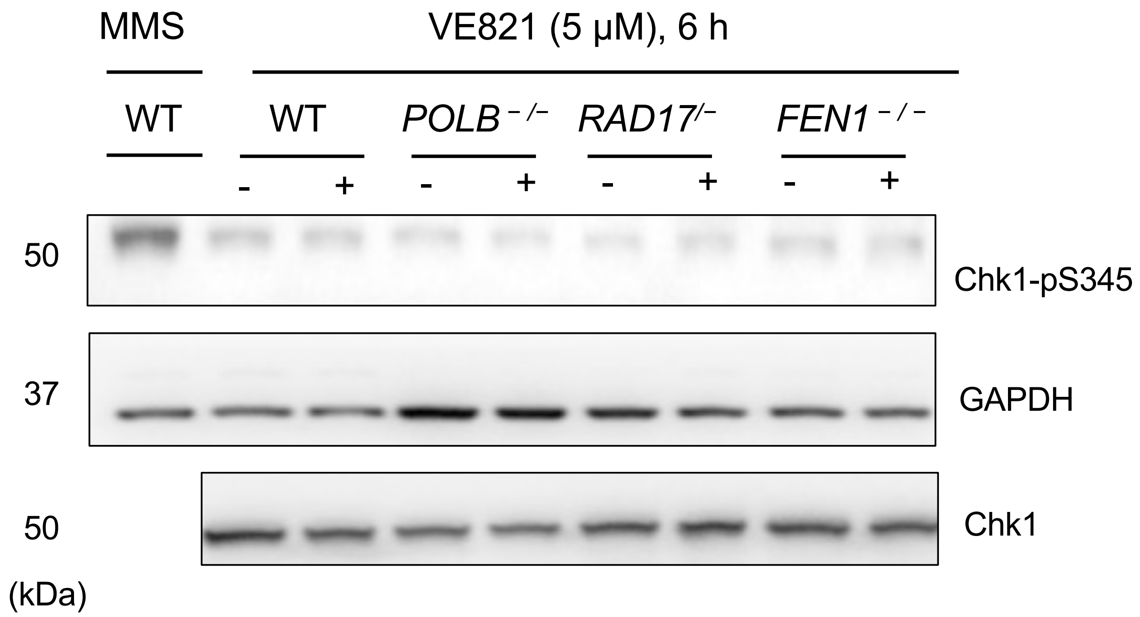

Figure S3 An ATR inhibitor VE-821 does not induce phosphorylation of Chk1.

Representative images of Western blot. Indicated DT40 cells were incubated with VE-821 (5 μM) for 6 h. Whole cell extracts were blotted for Chk1-pS345 (Chk1-p), Chk1, and GAPDH (loading control).

Fig. 1 raw images

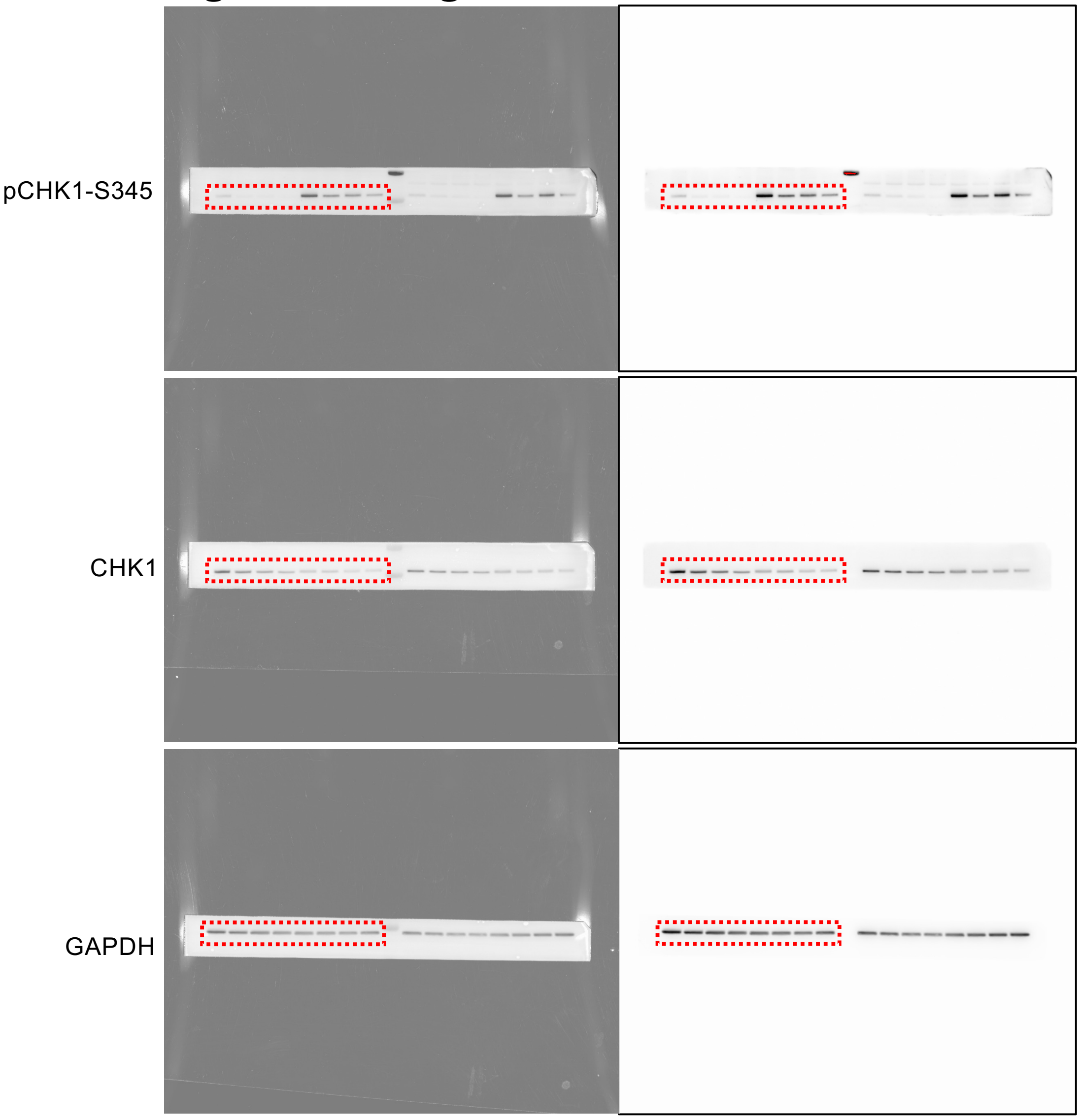

Fig. S3 raw images

pCHK1-S345

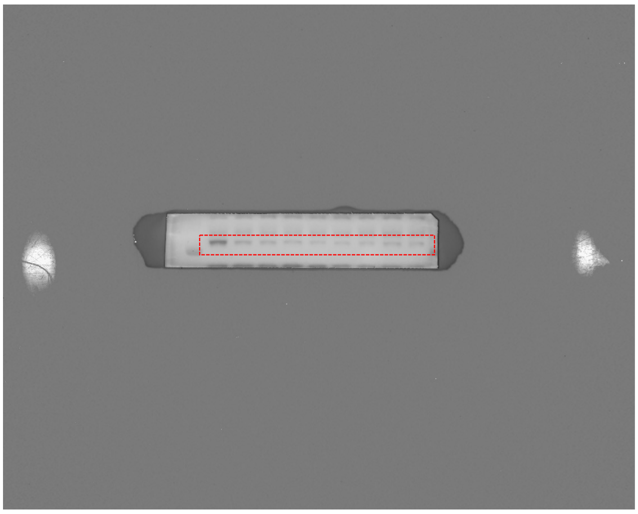

GAPDH

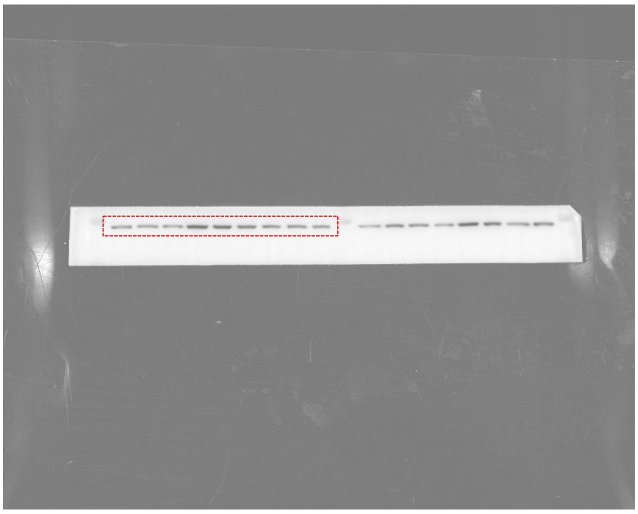

CHK1

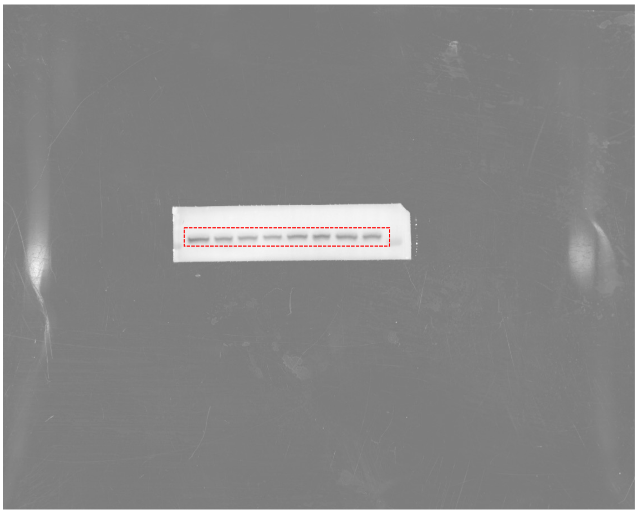

Figure S4 Uncropped images used in this study

Mass and NMR data: The HR-MS and NMR characterization data of **SPK67** and **SPK98** are available in previously published report [80]**.**
